# Association of high consumption of soy products with the risk of cognitive impairment and major neurocognitive disorders: a systematic review and dose-response meta-analysis

**DOI:** 10.3389/fnut.2025.1635844

**Published:** 2025-08-21

**Authors:** Jialin Yu, Hongmei Zeng

**Affiliations:** Department of Neurology, The Second Affiliated Hospital, Jiangxi Medical College, Nanchang University, Nanchang, China

**Keywords:** cognitive decline, cohort study, soy products, major neurocognitive disorder, dose-response meta-analysis

## Abstract

**Objective:**

While soy products can potentially affect cognitive function through various mechanisms, the dose-response connection of high soy consumption with major neurocognitive disorder or cognitive impairment remains unclear.

**Methods:**

A comprehensive retrieval was performed on PubMed, Embase, Cochrane Library, and Web of Science databases up to September 2024, to identify prospective or cohort studies (without language restrictions) examining the link between high soy consumption and the likelihood of developing major neurocognitive disorder or cognitive impairment. Stata (V15) was employed for data analysis, and a restricted cubic spline (RCS) model was employed for examining the dose-response effect.

**Results:**

Six studies incorporating 68,691 participants were included. Elevated consumption of total soy products was markedly correlated to a reduced likelihood of developing major neurocognitive disorder [odds ratios (OR) = 0.92, 95% confidence intervals (95%CI): 0.84–0.99]. While an association with increased risk of cognitive impairment was observed, it was not statistically significant. The dose-response meta-analysis indicated that a 1-g per day increase in the consumption of soy and natto demonstrated a correlation with an 8 and 14% decrease in the likelihood of developing major neurocognitive disorder, respectively. Subgroup analysis suggested a more pronounced protective effect in individuals not experiencing stroke (OR = 0.66, 95%CI: 0.53–0.82). However, soy consumption showed a paradoxical association with the likelihood of developing cognitive impairment (OR = 1.20, 95%CI: 0.83–1.72). Risk data showed no significant association with cognitive impairment. Dose-response data further explained an inverse relationship between dose and risk of cognitive impairment, with higher doses associated with lower risk.

**Conclusion:**

Elevated consumption of diverse soy products exhibited a linear negative correlation with cognitive decline or major neurocognitive disorder; however, significant heterogeneity remained within both the overall analysis and multiple subgroup analyses.

**Systematic Review Registration:**

https://www.crd.york.ac.uk/prospero/, CRD42024508555.

## 1 Background

As the global population ages rapidly, major neurocognitive disorder has emerged as one of the most significant global challenges for 21st-century healthcare and social care systems. Statistics suggest that the global prevalence of major neurocognitive disorder is projected to reach 78 million by 2030, and the associated economic burden is expected to surpass USD 2.8 trillion (1, 2). Given this scenario, primary prevention of major neurocognitive disorders is of paramount importance for reducing their societal burden. Evidence suggests that interventions targeting modifiable risk factors may delay or prevent major neurocognitive disorder in up to 40% of cases (3). A plausible link is observed between legume consumption and the incidence of major neurocognitive disorder. Soys, rich in nutrients and bioactive compounds, have been associated with reduced serum cholesterol and offer benefits against diabetes, hypertension, cardiovascular problems, and neuroinflammation. Notably, soy is an integral component of many traditional Asian diets (4–7). Epidemiological data indicate a lower major neurocognitive disorder prevalence in East Asia (4.2%) in comparison to Western nations, including the United States (6.5%) and Europe (6.9%). This disparity may be attributable to distinct dietary patterns prevalent in Asia, including the frequent and high consumption of legumes and soy products (8–11). Soybeans are rich in natural phytoestrogens. Soy products refer to foods primarily made from soy through various processing methods, including tofu, soy milk, tofu skin, and natto. These products might offer some protection against major neurocognitive disorder by reducing inflammation and oxidative stress, and by preventing *β*-amyloid-induced cellular apoptosis. Moreover, soybean-derived isoflavones have been implicated in the potential prevention of major neurocognitive disorder; however, (12, 13) other components of soy-based foods may exert adverse effects on cognitive function (14). To date, the potential link between soy product consumption and the likelihood of cognitive disorders has been explored in multiple research projects (15, 16). Findings from a prospective cohort study indicated a link between higher consumption of soy products and a lower likelihood of cognitive dysfunction (16, 17). Conversely, another investigation discovered that the overall consumption of soy product was not associated with the likelihood of disabling major neurocognitive disorder in either gender (16, 17). Soy proteins are rich in branched-chain amino acids (BCAAs; leucine, isoleucine, valine), which may influence cognitive function through multiple pathways (18). Emerging evidence suggests that: BCAAs modulate mTOR signaling and synaptic plasticity in the hippocampus; BCAA metabolism intersects with glutamate/GABA neurotransmitter cycling; Dysregulated BCAA levels are observed in Alzheimer’s disease patients (19, 20). While soy isoflavones (e.g., genistein, daidzein) have been the primary focus of neuroprotective research, emerging evidence highlights several additional bioactive components and pathways that may contribute to cognitive effects, such as BCAA metabolism, fermentation-derived bioactives, gut microbiome modulation, and multi-target neuroprotection (21, 22).

In addition, investigations have revealed a negative correlation of natto intake with the likelihood of developing disabling major neurocognitive disorder in females. However, no significant association was observed in males. Several factors may contribute to these disparate findings, including thyroid status, individual characteristics potentially affecting isoflavone bioavailability (involving age, sex, and race/ethnicity), and other study design factors (including the category of soy product consumed, isoflavone dose, frequency of dietary consumption, and the cognitive test instruments employed) (23). In summary, a meta-analysis will be carried out in this research to comprehensively evaluate the association of high soy product consumption with the likelihood of major neurocognitive disorder or cognitive decline. The research will examine the strength of these associations, assess potential dose-response relationships, and determine whether high soy product consumption is a protective factor against major neurocognitive disorder or cognitive impairment, thus providing high-quality evidence for primary major neurocognitive disorder prevention strategies.

## 2 Materials and methods

Registered prospectively with the PROSPERO under registration number CRD42024508555, the research protocol was developed in accordance with the PRISMA guidelines (24).

### 2.1 Literature retrieval

A comprehensive literature retrieval was conducted in PubMed, Cochrane Library, EMBase, and Web of Science Core. The retrieval covered all records from the inception of each database up to September 2024 (the last retrieval date). MeSH + free-text words were adopted during the retrieval process. The retrieval strategies, tailored to the specific characteristics of each database, were developed using a combination of free-text terms and MeSH: “major neurocognitive disorder,” “Alzheimer’s disease,” “Cognitive impairment,” “Cognitive Decline,” “isoflavone”“soy product,” “Dietary Fiber,” and “soybean.” All potentially eligible studies were included, irrespective of primary outcome or language. Furthermore, manual searches of the reference lists of key reviews published in English were performed. A representative search strategy, as implemented in PubMed, is provided in Appendix 1.

### 2.2 Eligibility criteria

Inclusion criteria: (1) Study design: This review included observational and prospective studies published without language restrictions. (2) Research participants: The study population consisted of adult participants aged 18 years or above. (3) Exposure factors: Dietary consumption of soy, soy-based products, or isolated isoflavones and studies employed validated food frequency questionnaires (FFQs) to quantify soy product intake, with intake levels reported as grams per day (g/day). The following three dietary assessment methods have been included: the Food Frequency Questionnaire (FFQ), the 24-h dietary review method, and the 3-day continuous dietary weight recording method. (4) Outcome measures: Cognitive impairment and major neurocognitive disorder status as determined by clinical diagnosis of major neurocognitive disorder, standardized cognitive impairment tools (such as MMSE, MoCA), and providing relative risks (RR)/odds ratios (OR) and associated 95% confidence intervals (95%CI) for calculations. Specific scales adopted are Brief Mental State Examination (MMSE), Wechsler Memory Scale-Revised (WMS-R) Logical Memory I/II Test, Clock Drawing Test, Clinical major neurocognitive disorder Rating Scale (CDR), Hasegawa’s major neurocognitive disorder Scale-Revised (HDS-R), Singapore Modified Mental State Examination (SM-MMSE), Alzheimer’s Disease Neuroimaging Initiative (J-ADNI) standardized tools, relative risks (RR)/odds ratios (OR) and associated 95% confidence intervals (95%CI) on the link between soy product consumption and the incidence of major neurocognitive disorder/Alzheimer’s disease/cognitive dysfunction (encompassing overall cognition, executive function, processing speed, attention, language, verbal memory, as well as visual memory). For specific details, please refer to Table 1. These measures will be extracted directly from the source publications or derived through calculations based on the original data presented.

**Table 1:** Other characteristics of studies included in the meta-analysis.

Authors	Published years	The dose of exposure	Exposure method	Methodology for assessing results	Dietary assessment methods	Adjustment for age	Adjustment for educational attainment
Utako Murai (23)	2022	142.5 g/d	Use of the validated Food Frequency Questionnaire (FFQ) from the 5-year follow-up survey conductd between 1995 and 1998	Determination of major neurocognitive disorder-related impairment of daily living status based on Japan’s Long-Term Care Insurance scheme	food frequency questionnaire (FFQ)-based method combined with food composition table estimation	In the Cox proportional risk regression model, age was adjusted as a continuous variable and analyses were stratified by age (<60 and ≥60 years).	Obtain information on educational attainment in Cohort I (middle school and below, high school, college and above)
Kazumasa Yamagishi (25)	2023	88.1 g/d	Adoption of the 24-h dietary recall method from 1985 to 1999	Determination of new-onset disabling major neurocognitive disorder based on Japan’s national long-term care insurance system	24-h dietary recall-based method	In the proportional risk regression model, age is included as a continuous variable in the adjustment	In the proportional risk regression model, which incorporates age as a continuous variable in the adjustment
Thomas Svensson (26)	2021	40.3 g/d	A 147-item semi-quantitative food frequency questionnaire (FFQ) from two surveys in 1995 and 2000 was used.	Determination based on the 2014–2015 Mental Health Screening combined with multiple scales and diagnostic criteria (Mini-Mental State Examination (MMSE), Wechsler Memory Scale-Revised (WMS-R) Logical Memory I/II subtests, Clock Drawing Test, and Clinical major neurocognitive disorder Rating (CDR))	semi-quantitative food frequency questionnaire (FFQ)-based method	In the logistic regression model, age was included as a continuous variable in the adjustment (Model 1 and subsequent models include age as a factor)	Categorization of educational attainment into junior high school, senior high school, university/vocational school/others
Rie Kishida (29)	2021	181.3 g/d	Adopting the 24-h dietary recall method between 1985 and 1999	Determination of disabling major neurocognitive disorder based on Japan’s national Long-Term Care Insurance (LTCI) system	24-h dietary recall-based method	The 24-h dietary recall method was used for the period 1985–1999	Categorize educational attainment into low (<7 years), medium (7–12 years) and high (≥13 years)

The following contents were excluded: (1) Publications lacking readily available full text or statistical information, such as OR, hazard ratio (HR), and 95%CI. (2) Animal tests (involving pharmacological or pharmacokinetic research). (3) Non-treatises (case reports, letters, abstracts and conference papers), reviews, and meta-analysis. (4) Duplicated publications. (5) Publications not in English.

### 2.3 Literature screening and data extraction

The titles and abstracts of the articles were independently examined by two researchers, and full texts were obtained for those meeting the pre-defined inclusive criteria. The researchers further cross-checked the articles. Discrepancies were settled by discussion until agreement was reached, or, if necessary, adjudicated by a third reviewer after a full-text review. Data sourced from each selected publication incorporated the first author, publication year, country, sample size, gender, age, follow-up duration, and soy product intake. The risk of bias was independently determined by two reviewers according to the PRISMA statement.

### 2.4 Literature quality evaluation

The quality of the included cohort studies was assessed by two independent researchers utilizing the Newcastle-Ottawa Scale (NOS). All disagreements were resolved by discussion with a third reviewer to reach consensus. The NOS scale comprises 8 items spanning 3 dimensions: selection of study participants, comparability, and outcome assessment. Each item is scored 1 point, with the exception of the comparability item, which is scored 2 points. A score exceeding 7 was indicative of low risk, a score between 5 and 7 inclusive represented moderate risk, and a score below 5 was considered to denote high risk.

### 2.5 Statistical analysis

Analyses of the data were performed using Stata (V15). ORs were employed as the effect size to quantify the link between soy product consumption and the outcome. These ORs were calculated by analyzing the highest versus the lowest (or a designated reference) level of soy product consumption. In cases where the literature provided effect sizes (ORs) for the relationship of cognitive impairment or major neurocognitive disorder with the intake of multiple types of (fermented or non-fermented) soy products, the OR value corresponding to the soy product type with the highest exposure in the population should be selected for analysis. If individual studies did not specify the number of individuals exposed to the highest dose of each type of soy product, a random-effects model is recommended for pooling the ORs across the studies. The I^2^ test was employed to examine statistical heterogeneity among the included publications. If the articles exhibited evidence of homogeneity (*p* ≥ 0.05 and *I*^2^ < 50%), a fixed-effects model was selected; otherwise (*p* < 0.05 or I^2^ ≥ 50%), a random-effects model was employed. Furthermore, potential factors contributing to the observed heterogeneity were examined through meta-regression and univariate sensitivity analyses. Subgroup analyses were carried out, where possible, based on follow-up duration, country, and study type (prospective or cohort studies) to understand potential causes of heterogeneity. For a more rigorous exploration of these sources, we excluded studies deemed to have a high risk of bias. Publication bias was assessed through Egger’s test and visual inspection of funnel plots.

A restricted cubic spline regression model using the glst function was employed to perform dose-response meta-analysis. A *p*-value less than 0.05 indicated the presence of a non-linear relationship between the variables, while a *p*-value ≥ 0.05 suggested a linear relationship.

## 3 Results

### 3.1 Literature screening procedure and results

**Figure 1:**
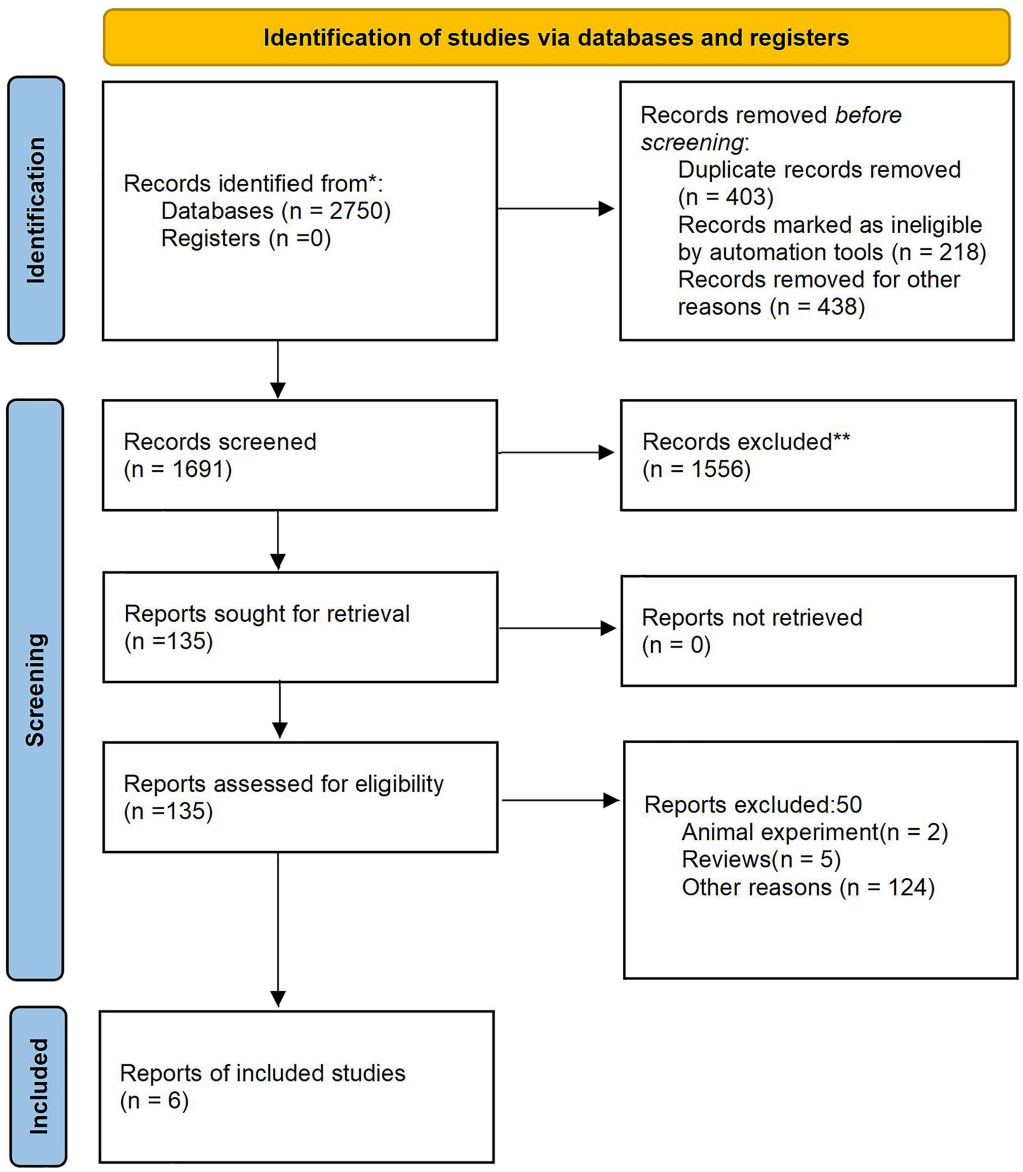
PRISMA flow diagram summarizing literature retrieval, study identification and selection.

A preliminary review of the database yielded 2,750 relevant articles, and a total of 1,691 articles were included. Following a comprehensive review of abstracts and full texts, 7 articles (16, 23, 25–29) meeting the inclusive criteria were ultimately selected. The literature screening process and results are depicted in Figure 1.

### 3.2 General characteristics and quality assessment results of the included literature

The analysis ultimately incorporated data from 68,691 individuals across 6 cohort studies. All studies were observational. The age of participants varied between 40 and 81 years. Participants in the adult health follow-up studies were aged ≥40 years. Seven studies were conducted in Asia (6 in Japan, 1 in Singapore). Participants were followed for a period of 4.7 to 18 years. The incidence of major neurocognitive disorder was assessed using the International Classification of Diseases (ICD) or the Diagnostic and Statistical Manual (DSM-IIIR/IV). All included articles were adjusted for confounders, and all six studies had NOS scores of ≥7 (Table 2). The highest intake level of soy, natto, and tofu assessed in relation to major neurocognitive disorder was 181.3 g/d. For the study evaluating soy consumption and disabling major neurocognitive disorder, the maximum intake examined was 141.8 g/d. The maximum intake of soy, tofu, and isoflavones observed in investigations related to cognitive impairment was 40.3 g/d.

**Table 2:** Basic characteristics of studies included in the meta-analysis.

Authors	Published years	Country	Sample size	Age	Study design	Exposure	Cognitive outcomes	Adjustments	Follow up
Utako Murai (23)	2022	Tokyo, Japan	41,447	45–74 y	a population-based prospective cohort study	Total soy product intake, Natto, Miso, Tofu, Isoflavone	disabling major neurocognitive disorder	age, body mass index, smoking status, alcohol intake, history of cancer, history of diabetes mellitus, medication for hypertension or hypercholesterolemia, metabolic equivalents	9.4 y
Kazumasa Yamagishi (25)	2023	Japan	3,739	40–64 y	an ongoing dynamic community cohort study	Beans	Total major neurocognitive disorder, major neurocognitive disorder with a history of stroke, major neurocognitive disorder without a history of stroke	Age and sex, body mass index, systolic blood pressure, antihypertensive medication use, serum total cholesterol, cholesterol-lowering medication, and diabetes	19.7 y
Thomas Svensson (26)	2021	Japan	1,036	40-59 y	a large population-based prospective cohort study	Isoflavone, Soy food, Tofu, Miso soup, Natto, Fermented soy (Natto + Miso)	Cognitive Impairment:	age, sex, education, and energy, adjusted folate intake, alcohol consumption, smoking status, physical activity, BMI, physical activity, use of prescribed medication, a history of diabetes mellitus, and a stroke, energy-adjusted intake of fish, meat, vegetables, fruits, and sodium.	5 or 10 y
Rie Kishida (29)	2021	Japan	3,739	40–64 y	a large population-based cohort	Beans, tofu, natto	disabling major neurocognitive disorder	age and sex, smoking status, drinking status, energy and fish intake	
Mohammad Talaei (27)	2019	Singapore	16,948	45–74 y	a population-based cohort study	Tofu equivalent, Isoflavones, Soy protein	cognitive impairment	age, sex, dialect, year of interview, educational level, and marriage status, body mass index, physical activity, smoking status, alcohol use, baseline history of self-reported hypertension, diabetes, heart attack, and stroke, history of cancer, sleep status, and total energy intake, dairy, red meat, poultry, fish, vegetables, fruits, tea, coffee, and soda, vegetable-fruit-soy (VFS) dietary pattern	20.2 y
Mariko Nakamoto (16)	2017	Japan	776	60–81 y	a prospective study	Total bean, Total soy product, Total soy product excluding, Soybean, Tofu, Deep-fried tofu, Natto, Soy sauce, Miso, Total isoflavone, Daidzein, Genistein, Glycitin	cognitive impairment	age and MMSE score at baseline and follow-up time, body mass index, duration of education, annual household income, medical history of ischemic heart diseases, diabetes, hypertension and hyperlipidemia, smoking, and energy intake at baseline	8.0 ± 3.0y (women) 7.7 ± 3.0y (men)

### 3.3 Results of the meta-analysis

#### 3.3.1 Association of high soy consumption with major neurocognitive disorder

**Figure 2:**
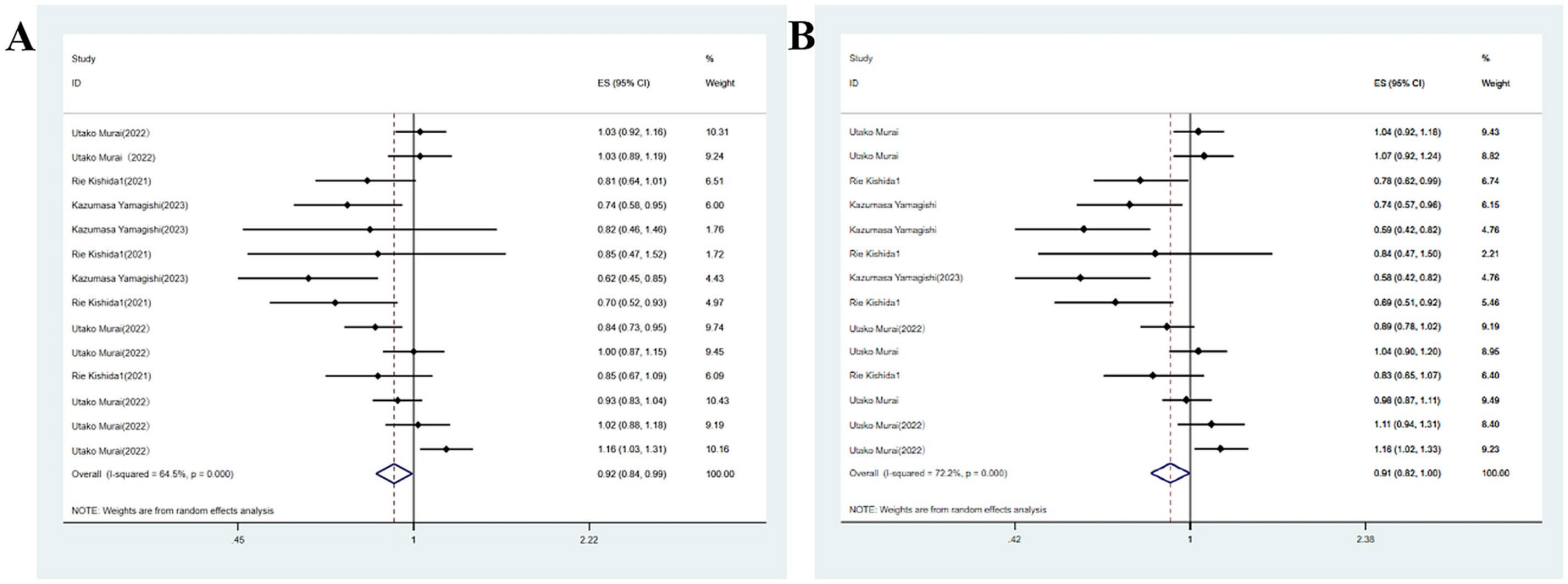
**(A)** Total soy intake and major neurocognitive disorder; **(B)** Total soy intake and neurocognitive disorder.

Studies on soy products such as soy and natto exhibited moderate heterogeneity (*I*^2^ = 64.5%, *p* < 0.05). Consequently, meta-analysis was conducted utilizing a random-effects model. The analysis revealed a link between higher soy product consumption and a decreased likelihood of developing major neurocognitive disorder (OR = 0.92, 95%CI: 0.84–0.99; Figure 2). The sensitivity analysis conducted by excluding studies one by one indicated that the effect size varied from the minimum (95% CI) to the maximum (95% CI), suggesting the robustness of the results. Multivariable analysis yielded similar results (OR = 0.91, 95%CI: 0.82–1.00), though statistical significance was not achieved.

#### 3.3.2 Association of high soy consumption with cognitive impairment

**Figure 3:**
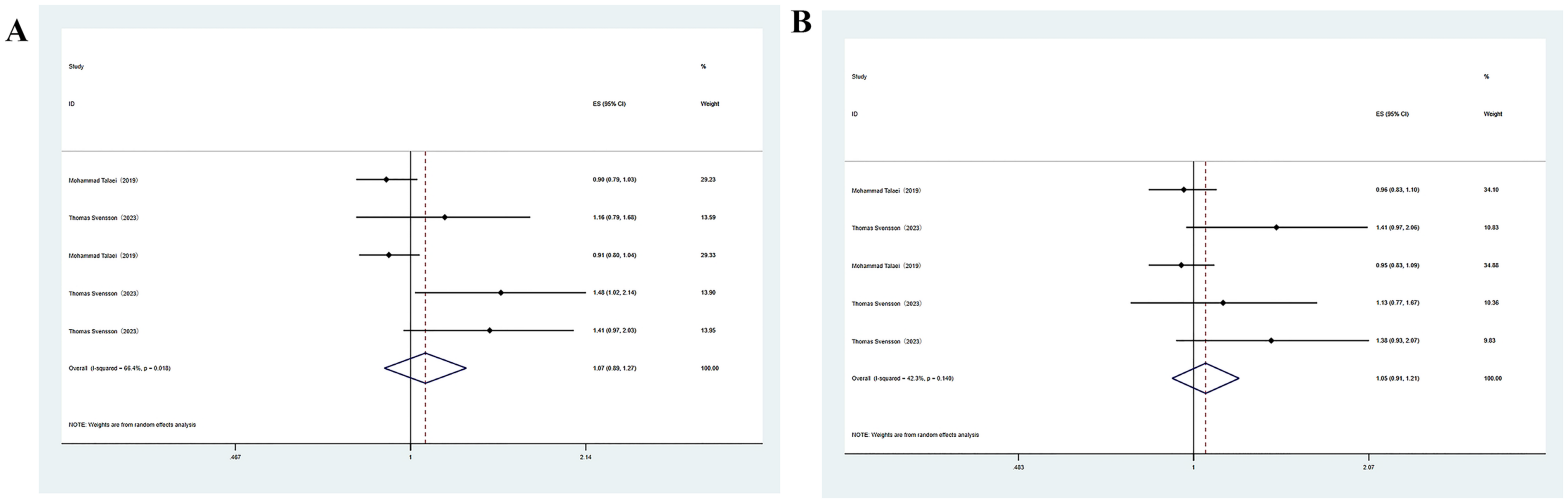
**(A)** Total soy products intake and cognitive impairment; **(B)** Total soy products intake and cognitive impairment.

The association of high soy consumption with cognitive impairment was reported in two studies. Given the substantial heterogeneity identified across publications, meta-analysis was carried out utilizing a random-effects model. The analysis revealed a link between high soy product consumption and an enhanced likelihood of developing cognitive impairment (Figure 3). Multivariable analysis yielded similar results (OR = 1.05, 95%CI: 0.91–1.21), though statistical significance was not achieved.

#### 3.3.3 Subgroup analysis

Participants were categorized into two subgroups based on their history of stroke. Meta-analysis using a random-effects model indicated that an association was observed between high soy consumption and a decreased likelihood of major neurocognitive disorder, regardless of stroke history. This association is notably more significant in individuals who have not experienced a stroke. Detailed results are presented in Table 3. Further subgroup analyses were performed to investigate the effect of specific soy product types. Results from two studies indicated that high intake of natto was negatively correlated with the incidence of disabling major neurocognitive disorder. In the analysis of the association of the tofu and natto consumption with the likelihood of developing major neurocognitive disorder, high intake of both tofu and natto was found to reduce the risk of major neurocognitive disorder. Notably, the research findings for natto were statistically significant. Moreover, higher intake of soy-derived isoflavones was associated with an enhanced likelihood of cognitive impairment. Similarly, dietary consumption of tofu was found to reduce the risk of cognitive impairment. A summary of the findings can be found in Table 4.

**Table 3:** Meta-analysis results of soy product intake and major neurocognitive disorder risk.

Univariate/multivariate analysis	Outcome measure/subgroup	Number of articles	I^2^ statistic for heterogeneity	OR	95%CI-LL	95%CI-UP	*p*
Univariate analysis							
Soy	Patients with a history of stroke	2	0.0%	0.83	0.55	1.26	0.39
	Patients without a history of stroke	2	0.0%	0.66	0.53	0.82	0.00
	Disabling major neurocognitive disorder	2	45.7%	0.98	0.87	1.11	0.75
	Tofu	2	0.0%	0.94	0.87	1.03	0.17
	Natto	2	21.8%	0.86	0.77	0.97	0.01
Multivariate analysis							
Soy	Patients with a history of stroke	2	0.0%	0.86	0.56	1.32	0.49
	Patients without a history of stroke	2	0.0%	0.64	0.52	0.80	0.00
	Disabling major neurocognitive disorder	2	63.4%	0.98	0.84	1.14	0.98
	Tofu	2	15.3%	0.98	0.89	1.08	0.66
	Natto	2	53.5%	0.89	0.76	1.04	0.15

**Table 4:** Meta-analysis results of soy products intake and risk of cognitive impairment.

Univariate/multivariate analysis	Outcome measure/subgroup	Number of articles	I^2^ statistic for heterogeneity	OR	95%CI-LL	95%CI-UP	*p*
Univariate analysis	Soy	2	79.2%	1.20	0.83	1.72	0.33
	Tofu	2	35.4%	0.96	0.77	1.19	0.67
	Isoflavone	2	64.7%	1.10	0.88	1.99	0.28
Multivariate analysis	Soy	2	64.7%	1.17	0.88	1.57	0.28
	Tofu	2	0.0%	0.97	0.85	1.10	0.53
	Isoflavone	2	79.0%	1.15	0.74	1.80	0.53

#### 3.3.4 Results of dose-response meta-analysis

**Figure 4:**
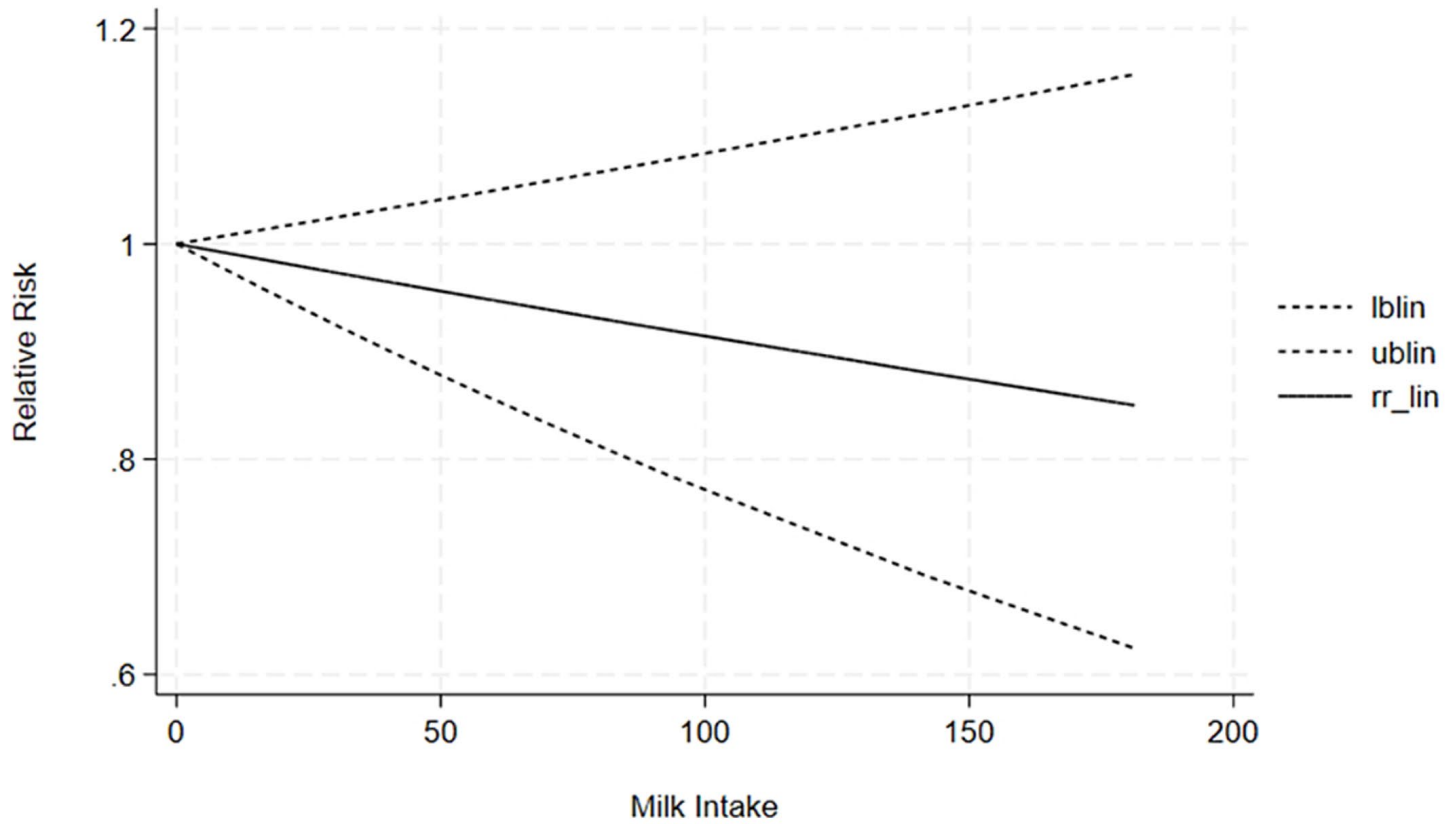
Dose-response correlation of soy intake with major neurocognitive disorder.

**Figure 5:**
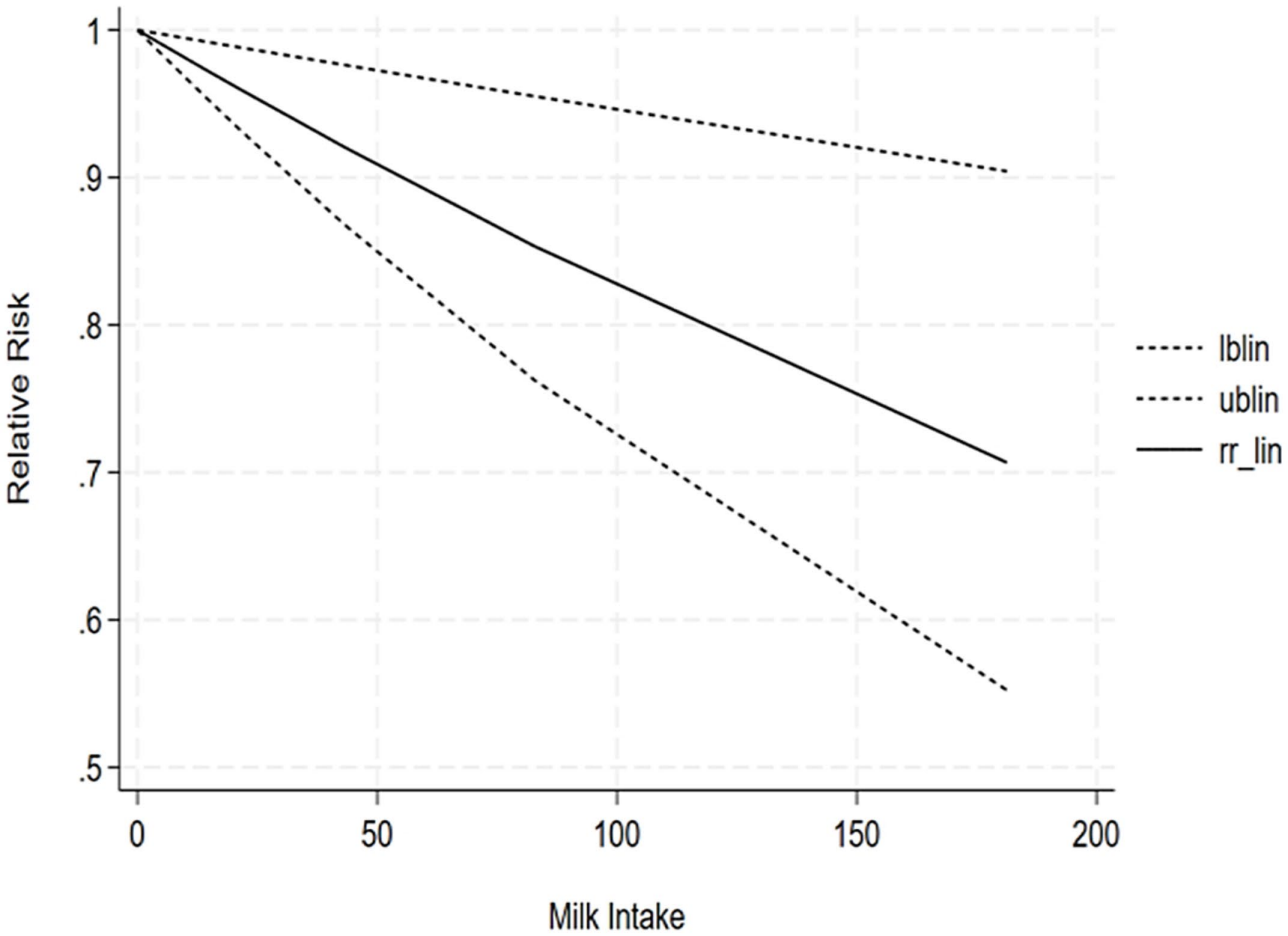
Dose-response correlation of natto intake with disabling major neurocognitive disorder.

**Figure 6:**
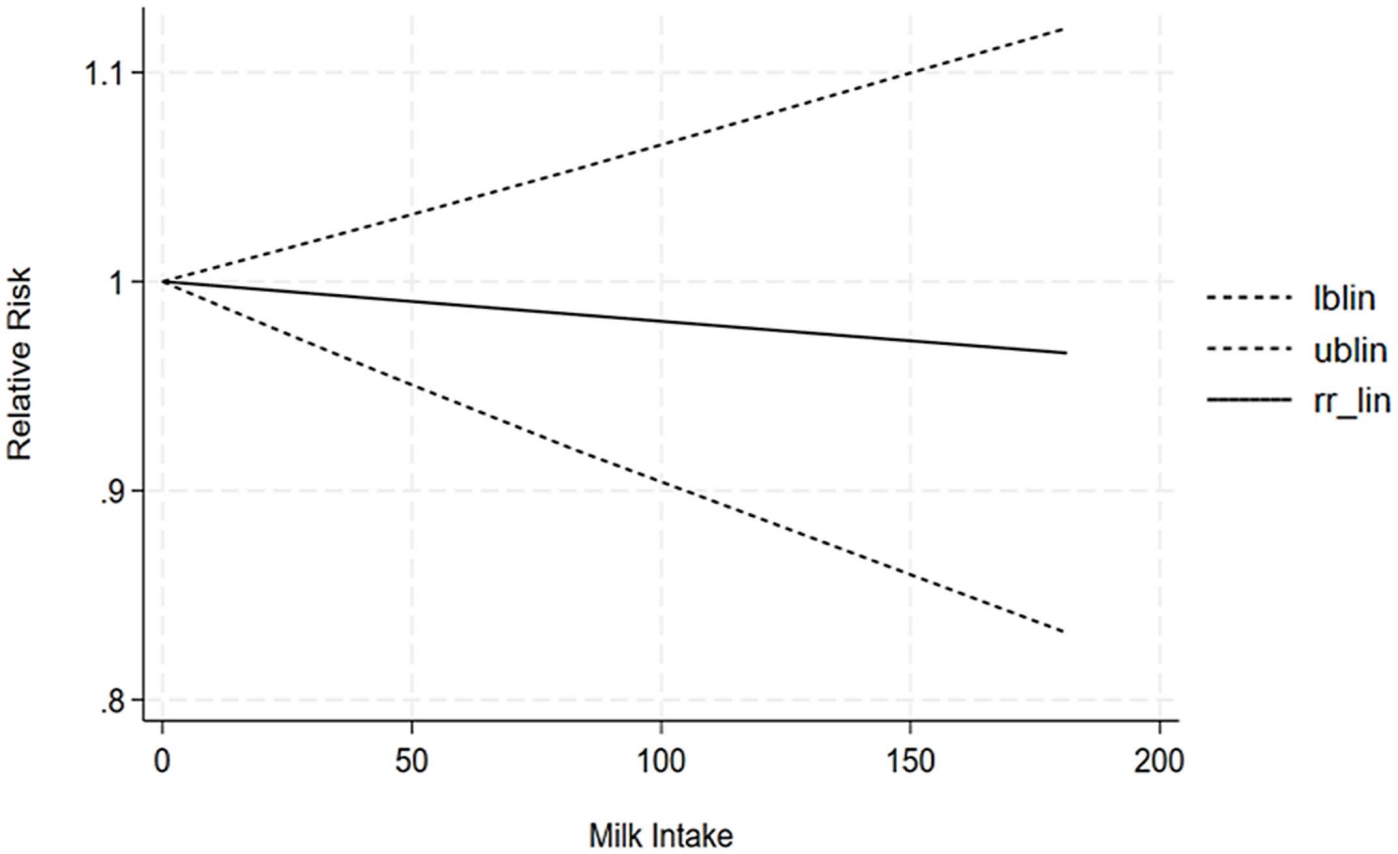
Dose-response correlation of tofu intake with major neurocognitive disorder.

The correlation between soy intake and the risk of major neurocognitive disorder was assessed utilizing a dose-response meta-analysis based on the findings of three studies, as depicted in Figure 4. An increase in soy intake of 1 g/day demonstrated a marked 8% reduction in the likelihood of developing major neurocognitive disorder (RR = 0.92; 95%CI: 0.84–0.99). With each 1 g/day increment in intake, the likelihood of disabling major neurocognitive disorder was markedly reduced by 14% for natto (RR = 0.86; 95%CI: 0.77–0.97) and by 6% for tofu (RR = 0.90; 95%CI: 0.87–1.03). The dose-response meta-analyses are presented in Figures 5, 6.

**Figure 7:**
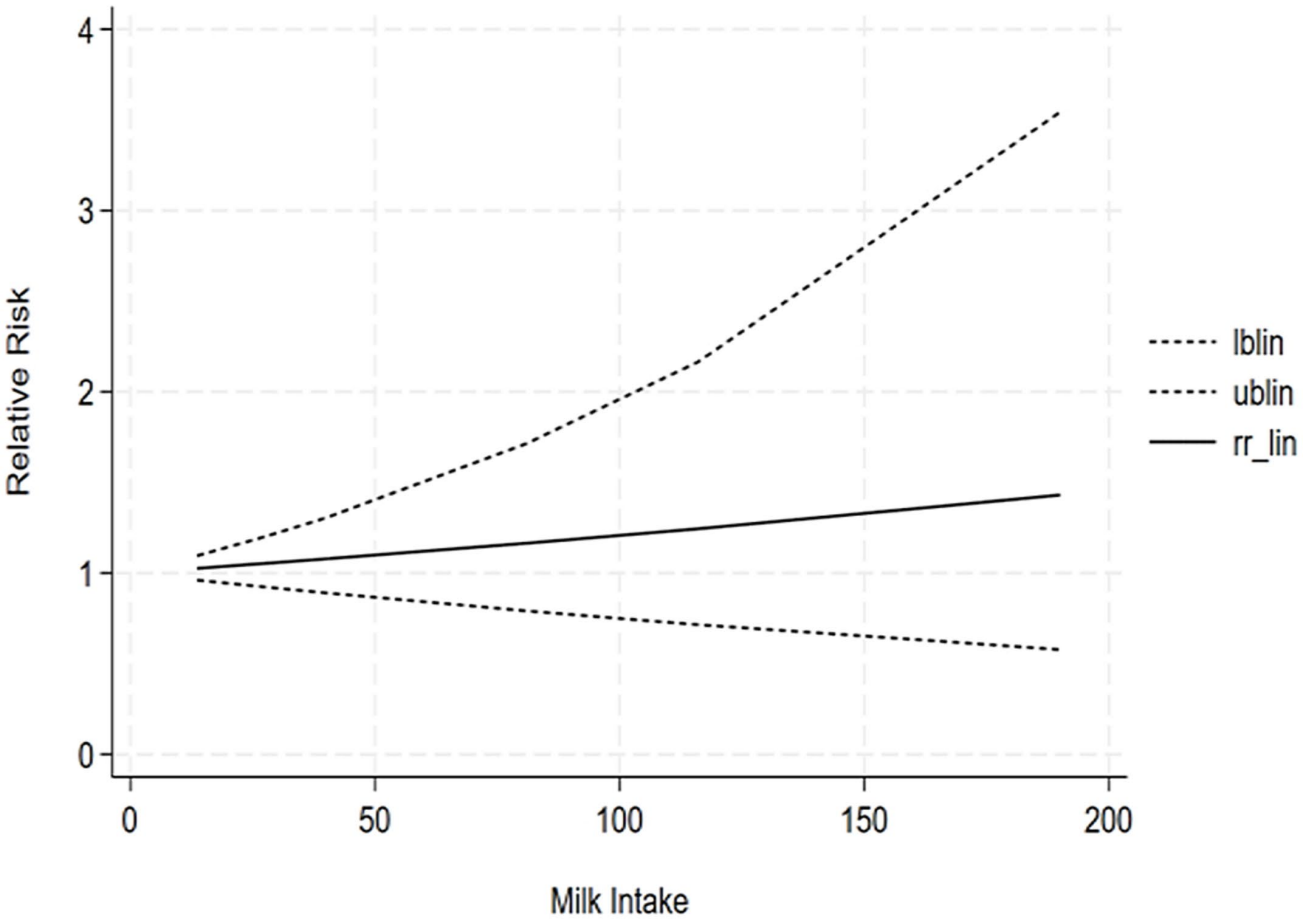
Dose-response correlation of tofu intake with cognitive impairment.

The correlation between tofu intake and the risk of cognitive impairment was assessed utilizing a dose-response meta-analysis based on the findings of two studies, as depicted in Figure 7. An increment in tofu intake of 1 g/day demonstrated a marked 4% decrease in the likelihood of developing cognitive impairment (RR = 0.96; 95%CI: 0.77–1.19). The likelihood ratio tests revealed a positive linear association of tofu consumption with the likelihood of developing cognitive impairment.

#### 3.3.5 Sensitivity analysis and publication bias

Sensitivity analysis was carried out utilizing a leave-one-out approach, and the results revealed no significant changes, suggesting relatively robust findings. Details of the sensitivity analysis are provided in Supplementary Figure 1. The funnel plot exhibited a generally symmetrical distribution, indicating a low likelihood of publication bias. Additionally, Egger’s test (*t* = 0.35, *p* = 0.730) and Begg’s test (*Z* = 0.25, *p* = 0.805) both exhibited no evidence of publication bias. The details are provided in Supplementary Figure 2.

## 4 Discussion

The research revealed that high intake of total soy products was markedly linked to a reduced likelihood of developing major neurocognitive disorder, with the association being more pronounced in individuals without history of stroke. This findings support the hypothesis proposed in previous studies that the Asian dietary patterns may decrease the risk of major neurocognitive disorder (29). It is noteworthy that fermented soy products, such as natto, demonstrated a comparatively more pronounced protective association. This may be related to their abundance of active constituents such as vitamin K2 and nattokinase, which have been shown to possess antithrombotic and anti-inflammatory effects (30). Despite inconsistent findings regarding the effects of soy products on cognitive impairment, overall soy consumption has been associated with an elevated risk. The factors contributing to this variability can be elucidated from multiple perspectives.

A study published in The Lancet in 2024 showed that modifying risk factors and lifestyle could prevent or delay 40% of AD and related major neurocognitive disorder, highlighting the necessity of focusing on modifiable risk factors for AD (3). Effective dietary approaches can enhance cognitive function and prognosis in older adults, which would provide broader context for soy as part of comprehensive dietary strategies (31). Williams et al. noted that cognitive training, Mediterranean diet, and Omega-3 fatty acids could delay or reduce the risk of developing major neurocognitive disorder (32). While our study focused specifically on soy products, emerging evidence suggests that cognitive protection in older adults is best achieved through synergistic dietary patterns rather than isolated nutrients. Recent studies have highlighted that the MIND diet (which includes soy as a component of legume intake) demonstrates stronger neuroprotective effects than any single food group, and has been shown to reduce the risk of major neurocognitive disorder by 53% in longitudinal cohorts. Fermented soy products (e.g., natto, miso) may complement other brain-healthy foods (e.g., leafy greens, berries) by enhancing gut microbiota diversity—a key mediator of the diet-cognition relationship. Combined interventions (e.g., soy isoflavones + omega-3 s) demonstrated additive benefits on hippocampal volume in trials, suggesting that our observed effects could be amplified in nutrient-diverse diets (33).

The research findings are consistent with those of some cohort studies. For instance, Utaro Murai et al. (23) found that natto consumption was linked to a decreased likelihood of developing disabling major neurocognitive disorder in a Japanese population, whereas Kazumasa Yamagishi (25) found no marked link between total soy product intake and the incidence of major neurocognitive disorder. The primary reasons for discrepancies in research findings include: (1) Differences in study subject characteristics: Age (elderly vs. middle-aged), gender (metabolic capacity variations), and regional ethnic characteristics (e.g., traditional Japanese soy diet vs. Western-influenced Singaporean Chinese diet). The Japanese population consumes a higher average daily soy intake (60 g), with natto and tofu as staple foods (29). (2) Variations in outcome definitions: Cognitive impairment is primarily assessed for mild cognitive decline using the MMSE scale, while major neuropsychiatric disorders focus on functional impairments (as defined by Japan’s long-term care insurance system), leading to differences in assessment dimensions. (3) Characteristics of soy products: Fermented products like natto may exhibit protective effects due to their unique composition and bioavailability, whereas methodological differences between assessment methods (3-day dietary records vs. food frequency questionnaires) could introduce bias. Other factors including confounding controls (e.g., education level, dietary structure), mechanisms of action (estrogen-like effects vs. vascular protection), study design (follow-up duration, sample size), and individual gut microbiota differences in isoflavone metabolism (e.g., stronger metabolic capacity in Asian populations) may all lead to inconsistent results (34).

Soy products may exert neuroprotective effects via the following pathways: (1) Bidirectional modulation of isoflavones. Acting as phytoestrogens, soy isoflavones (e.g., genistein) may inhibit *β*-amyloid deposition by activating ERβ receptors and promote synaptic plasticity within the hippocampus (34–37). Nonetheless, excessive consumption of isoflavones may disrupt thyroid function, potentially exacerbating cognitive decline, especially in individuals with insufficient iodine intake (38). (2) Anti-inflammatory and antioxidant actions. Pyrazines derived from Natto and polyphenols extracted from tofu can inhibit the NF-κB pathway, leading to reduced levels of neuroinflammatory markers such as IL-6 and TNF-*α* (39). (3) Gut-brain axis modulation. Probiotics (e.g., *Bacillus subtilis*) in fermented soy products may improve blood-brain barrier function by modulating short-chain fatty acids (SCFAs) (40, 41).

Our findings demonstrated a linear dose-response relationship between soy intake and reduced risk of major neurocognitive disorder, wherein each 1 g/day increase was associated with an 8% lower risk, while highlighting important nuances regarding optimal consumption levels. The most significant protective effects were observed at moderate intake levels of 50–100 g/day. Notably, fermented products like natto showed a stronger 14% risk reduction per 1 g/day, which is comparable to the intake provided by approximately 1–2 daily servings of traditional soy foods. However, the current evidence reveals substantial variability in consumption patterns and study methodologies, with “high intake” definitions ranging from 40 to 180 g/day across different populations and soy products. The benefits appear more pronounced in older adults and plateau at intake levels exceeding 100 g/day, suggesting diminishing returns from increased intake. While these observations support the cognitive benefits of moderate, regular soy consumption - especially fermented varieties - they also underscore the need for more standardized research to establish definitive optimal dosing guidelines. Practical implementation should consider cultural dietary patterns, individual tolerance, and product-specific characteristics, with current evidence supporting 1–2 daily servings of traditional soy products as a reasonable approach for cognitive protection within balanced dietary patterns. Future studies should focus on age-stratified clinical trials and investigate potential synergistic effects with other neuroprotective foods to refine these recommendations further. Based on the results of the highest effective dose range in the dose-response analysis, we have supplemented the following practical recommendations: “Combined with existing research data, for people who want to reduce the risk of cognitive impairment by consuming soy through diet, it is recommended to consume about 190 g of soy products (such as tofu, natto, etc.) daily (measured as tofu equivalent). Future randomized controlled trials (RCTs) should focus on the exploration of the maximum effective dose of soybean intervention for cognitive impairment/severe cognitive impairment, which is important to clarify the dose-effect relationship and optimize the intervention protocol.

Based on existing evidence, soy demonstrates potential benefits for postmenopausal women and specific metabolic phenotypes of homo sapiens populations such as equol producers. The WHO supports that a healthy dietary pattern (increased plant-based food intake) can reduce chronic disease risks, thereby indirectly influencing cognitive function. The Alzheimer’s Disease Neuroimaging Initiative (ADNI) recognizes that the Mediterranean-DASH Intervention for Neurodegenerative Delay (MIND) diet (including legumes) may reduce the risk of cognitive decline (42, 43). The recommended daily intake of 15–25 g soybeans for adults, incorporated into a plant-based diet with whole grains, dark vegetables, and nuts, enhances cognitive protection through nutritional synergy. Conducting randomized controlled trials targeting specific populations (e.g., APOEε4 carriers and different ethnic groups) will clarify the “effective dosage” and “safety threshold” of soybean consumption. Exploring cognitive effect differences between subtypes of soy products (e.g., fermented vs. non-fermented varieties) will provide evidence-based guidance for more precise dietary recommendations. Individual differences in isoflavone metabolism should be noted to improve bioavailability. Approximately 30–50% of the population are “estrone producers,” individuals whose gut microbiota can convert isoflavones into more active estrone. Their cognitive protective effects may be more pronounced. Differences in metabolic enzymes (such as COMT) and estrogen receptor genotypes can influence the metabolic efficiency and signaling pathways of isoflavones. Soy products, as a low-cost and easily accessible dietary component, warrant attention for their cost-effectiveness potential in the field of cognitive health. Future research can employ cohort study data modeling to analyze the association between soy product intake and cognitive-related medical costs, or conduct community intervention trials to compare the costs and cognitive improvement effects of different soy product intervention strategies, thereby providing more concrete economic evidence for practical applications.

Our research findings carry important clinical implications for integrating soy products into prevention strategies for major neurocognitive disorder. The observed dose-response relationship—with each 1 g/day increase in soy intake associated with an 8% reduction in the risk of major neurocognitive disorder—suggests that moderate dietary modifications may yield meaningful benefits, particularly in Asian populations where traditional soy consumption patterns are already established. The stronger protective effects observed in fermented products like natto (14% risk reduction per 1 g/day) highlight the potential value of prioritizing these varieties in clinical recommendations, especially for patients with vascular risk factors who may benefit from nattokinase’s fibrinolytic properties. However, practitioners should remain cognizant of individual variability. The attenuated effects observed in stroke survivors and the neutral associations found with cognitive impairment in middle-aged adults suggest that soy’s benefits may be most pronounced for primary prevention in older, neurologically healthy individuals. These findings complement existing dietary guidelines advocating plant-based proteins while emphasizing the importance of considering food form (fermented vs. non-fermented) and cultural dietary contexts. Clinicians recommending increased soy intake should consider potential thyroid interactions in vulnerable patients and implement gradual dietary modifications rather than abrupt changes. The relatively modest effect sizes reinforce that soy should be viewed as one component of a comprehensive brain-healthy diet rather than a standalone intervention, consistent with contemporary nutritional approaches that emphasize dietary pattern synergy over the effects of isolated foods (44). Future clinical trials should explore whether combining soy with other neuroprotective nutrients (e.g., omega-3 or polyphenol-rich foods) might yield additive benefits for cognitive aging (45).

### 4.1 Limitations

1. Geographic bias: As all included studies were conducted in Asian populations (6 in Japan and 1 in Singapore), caution is warranted when generalizing these findings to individuals of other ethnicities and geographic regions.
2. Heterogeneity: A moderate I^2^ value of 65.1% was observed, mainly due to the following points: (1) Study subject differences: wide age ranges and ethnic variations; (2) Differences in gut microbiota metabolism of soy isoflavones across ethnic groups; (3) Variations in soy exposure types (soy protein, isoflavones, soy products), dosages, and follow-up durations; (4) Differences in outcome measures: varying diagnostic criteria for severe cognitive impairment (clinical diagnosis vs. scale scores) may affect the sensitivity to cognitive function. (5) Differences in control and analysis methods: varying degrees of adjustment for confounding factors (e.g., education level, age) and statistical models. (6) Implications of heterogeneity: High heterogeneity in pooled effect sizes indicates a reflection of more “average trends,” necessitating caution when extrapolating to different populations or exposure patterns, and potentially compromising the clarity of causal inference. (7) Results were pooled using random effects models to reduce the risk of bias; subgroup analyses by stroke history and soy type (e.g., “a stronger association with higher soy intake was observed in stroke-free populations, while natto consumption demonstrated greater statistical significance”) partially explained the heterogeneity; sensitivity analysis revealed stable result directions but fluctuating magnitudes, which were influenced by heterogeneity.
3. Residual confounding: Despite adjustments for basic metabolic indices in most studies, socioeconomic status, genetic factors (e.g., APOE ε4), and comprehensive dietary habits may still have affected the findings. The included studies exhibited heterogeneity in their classification and analysis of different soy products. While some studies examined specific products like natto or tofu individually, others reported only combined ‘total soy product’ intake. This heterogeneity in exposure assessment, particularly the grouping of products with potentially distinct biological effects (e.g., fermented vs. non-fermented, minimally processed vs. ultra-processed), may introduce bias and restrict the ability to draw product-specific conclusions. Unfortunately, the limited number of studies precluded meaningful subgroup analyses by product type or processing level. Future studies should focus on more standardized and detailed report for specific soy product exposures.
4. Insufficient dose-response data: Only three studies provided continuous dose-response data, limiting our ability to infer an optimal intake level.

### 4.2 Future research directions

1. Cross-cultural cohort studies: these studies are warranted to validate the association of soy product consumption with cognitive function in Western populations, and to compare the heterogeneity of effects across diverse dietary backgrounds.
2. Mechanistic exploration: The underlying mechanisms will be explored by conducting metabolomic analysis to investigate the dynamic relationships between bioactive isoflavone metabolites (e.g., equol) and cognitive biomarkers (e.g., plasma Aβ42/40 ratio).
3. Clinical trial design: Intervention trials should be conducted in high-risk populations (e.g., individuals with MCI) to determine the neuroprotective effects of specific soy product types and dosages (e.g., daily natto consumption of ≥50 g).
4. Policy recommendation: Cost-effectiveness analyses should be carried out to evaluate the feasibility of incorporating soy products into public health dietary guidelines (e.g., WHO guidelines for the prevention of major neurocognitive disorder) (46).

## 5 Conclusion

While acknowledging the presence of heterogeneity and limitations, our findings suggest that a higher consumption of soy and soy products may represent a potential dietary strategy for the prevention of major neurocognitive disorder. Further investigations should prioritize well-designed studies to clarify the dose-response correlation and underlying mechanisms, ultimately informing personalized nutritional interventions.

## Data Availability

The original contributions presented in the study are included in the article/Supplementary material, further inquiries can be directed to the corresponding author.

## Supplementary material

SUPPLEMENTARY FIGURE 1 Sensitivity analysis.

SUPPLEMENTARY FIGURE 2 Egger/Begg test.
