# Supplementary figures and images for: Association of high consumption of soy products with the risk of cognitive impairment and major neurocognitive disorders: a systematic review and dose-response meta-analysis
Source: Front Nutr. 2025 Aug 21;12:1635844. doi: 10.3389/fnut.2025.1635844 (PMC12408264; doi:10.3389/fnut.2025.1635844)

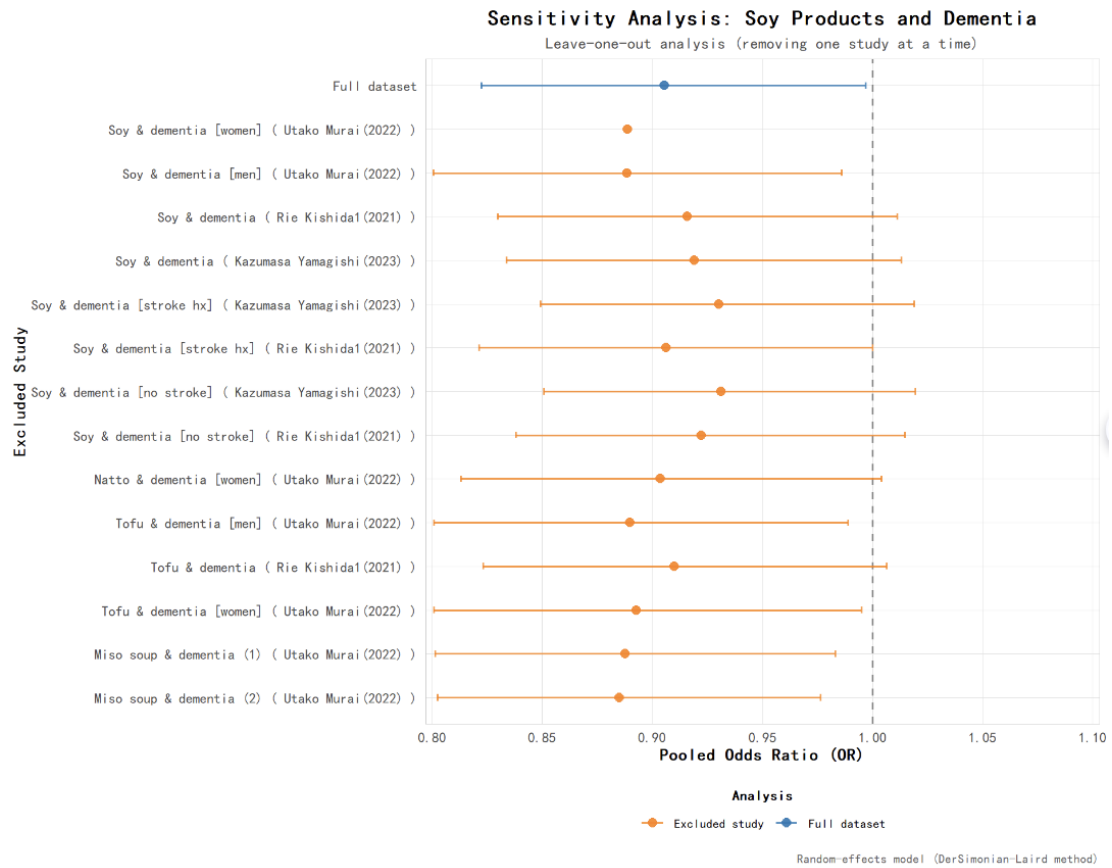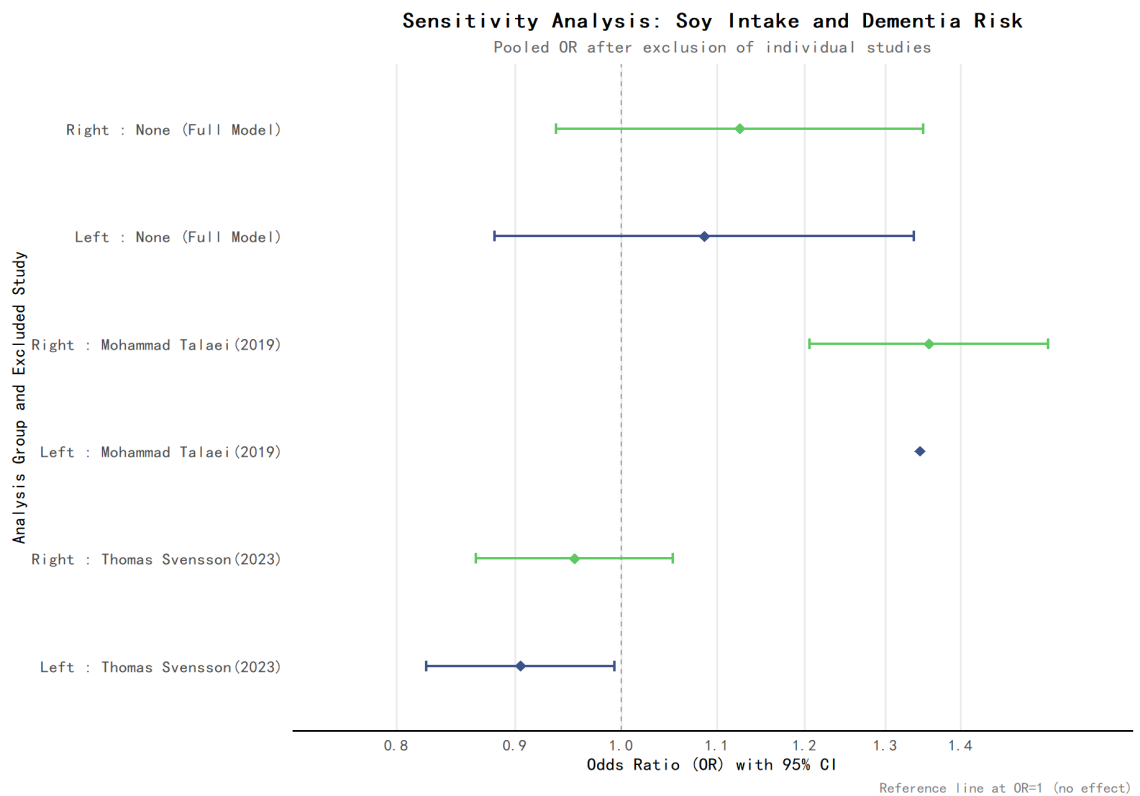

Supplement: SUPPLEMENTARY FIGURE 1 — Sensitivity analysis. [file Image_1.pdf]

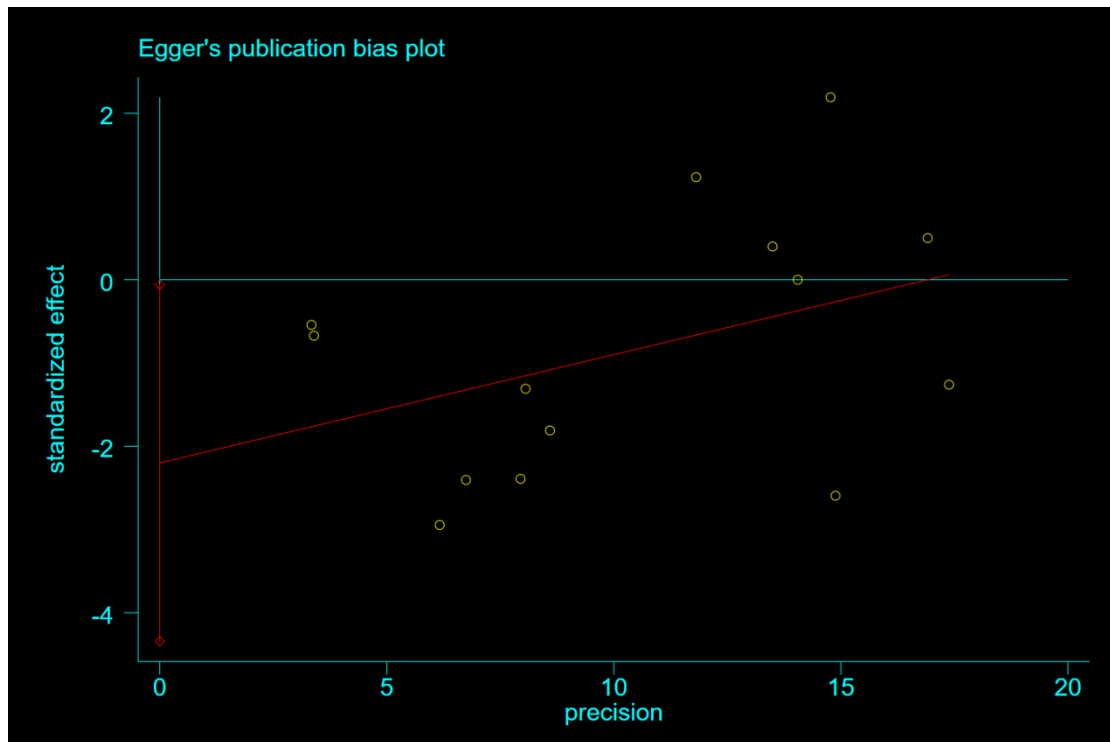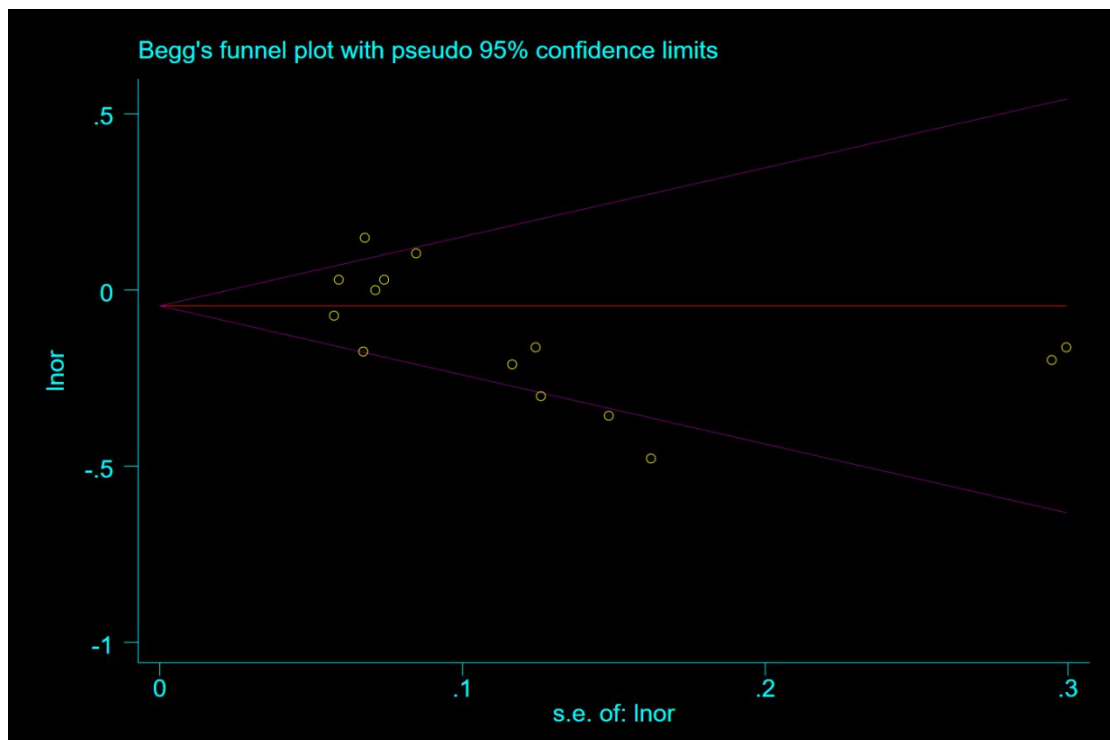

Supplement: SUPPLEMENTARY FIGURE 2 — Egger/Begg test. [file Image_2.pdf]
