# Supplementary material for: Association of high consumption of soy products with the risk of cognitive impairment and major neurocognitive disorders: a systematic review and dose-response meta-analysis
Source: Front Nutr. 2025 Aug 21;12:1635844. doi: 10.3389/fnut.2025.1635844 (PMC12408264; doi:10.3389/fnut.2025.1635844)
Supplement: Supplementary file 3 [file Table_1.pdf]

Appendix 1: Search strategy

((((("Dementia"[Mesh]) OR (((((((((((Dementias[Title/Abstract]) OR (Amentia[Title/Abstract])) OR (Amentias[Title/Abstract])) OR (Senile Paranoid Dementia[Title/Abstract])) OR (Dementias, Senile Paranoid[Title/Abstract])) OR (Paranoid Dementia, Senile[Title/Abstract])) OR (Paranoid Dementias, Senile[Title/Abstract])) OR (Senile Paranoid Dementias[Title/Abstract])) OR (Familial Dementia[Title/Abstract])) OR (Dementia, Familial[Title/Abstract])) OR (Dementias, Familial[Title/Abstract])) OR (Familial Dementias[Title/Abstract]))) OR (("Alzheimer Disease"[Mesh]) OR (((((((((((((((Cognitive Dysfunctions[Title/Abstract]) OR (Dysfunction, Cognitive[Title/Abstract])) OR (Dysfunctions, Cognitive[Title/Abstract])) OR (Cognitive Impairments[Title/Abstract])) OR (Impairment, Cognitive[Title/Abstract])) OR (Cognitive Disorder[Title/Abstract])) OR (Disorder, Cognitive[Title/Abstract])) OR (Mild Cognitive Impairment[Title/Abstract])) OR (Cognitive Impairment, Mild[Title/Abstract])) OR (Impairment, Mild Cognitive[Title/Abstract])) OR (Mild Cognitive Impairments[Title/Abstract])) OR (Cognitive Decline[Title/Abstract])) OR (Decline, Cognitive[Title/Abstract])) OR (Mental Deterioration[Title/Abstract])) OR (Deteriorations, Mental[Title/Abstract])) OR (Mental Deteriorations[Title/Abstract])))) OR (("Cognition"[Mesh]) OR (((((Cognitions[Title/Abstract]) OR (Cognitive Function[Title/Abstract])) OR (Cognitive Functions[Title/Abstract])) OR (Function, Cognitive[Title/Abstract])) OR (Functions, Cognitive[Title/Abstract])))) OR (("Cognitive Dysfunction"[Mesh]) OR (((((((((((((((Cognitive Dysfunctions[Title/Abstract]) OR (Dysfunction, Cognitive[Title/Abstract])) OR (Dysfunctions, Cognitive[Title/Abstract])) OR (Cognitive Impairments[Title/Abstract])) OR (Impairment, Cognitive[Title/Abstract])) OR (Cognitive Disorder[Title/Abstract])) OR (Disorder, Cognitive[Title/Abstract])) OR (Mild Cognitive Impairment[Title/Abstract])) OR (Cognitive Impairment, Mild[Title/Abstract])) OR (Impairment, Mild Cognitive[Title/Abstract])) OR (Mild Cognitive Impairments[Title/Abstract])) OR (Cognitive Decline[Title/Abstract])) OR (Decline, Cognitive[Title/Abstract])) OR (Mental Deterioration[Title/Abstract])) OR (Deteriorations, Mental[Title/Abstract])) OR (Mental Deteriorations[Title/Abstract])))) AND ((("Glycine max"[Mesh]) OR ((((((Soy Beans[Title/Abstract]) OR (Bean, Soy[Title/Abstract])) OR (Beans, Soy[Title/Abstract])) OR (Soy Bean[Title/Abstract])) OR (Soybeans[Title/Abstract])) OR (Soybean[Title/Abstract]))) OR (("Soy Foods"[Mesh]) OR ((((((((((((((◎Food, Soy[Title/Abstract]) OR (Soy Food[Title/Abstract])) OR (Texturized Soy Protein[Title/Abstract])) OR (Protein, Texturized Soy[Title/Abstract])) OR (Soy Protein, Texturized[Title/Abstract])) OR (Texturized Soy Proteins[Title/Abstract]))) OR (Soy Cheese[Title/Abstract])) OR (Cheese, Soy[Title/Abstract])) OR (Soy Cheeses[Title/Abstract])) OR (Soy Sauce[Title/Abstract])) OR (Bean Curd, Soy[Title/Abstract])) OR (Tofu[Title/Abstract])) OR (Curd, Soy Bean[Title/Abstract])) OR (Soy Bean Curds[Title/Abstract]))))
